# Roles of IFN-γ and γδ T Cells in Protective Immunity Against Blood-Stage Malaria

**DOI:** 10.3389/fimmu.2013.00258

**Published:** 2013-08-29

**Authors:** Shin-Ichi Inoue, Mamoru Niikura, Shoichiro Mineo, Fumie Kobayashi

**Affiliations:** ^1^Department of Infectious Diseases, Kyorin University School of Medicine, Mitaka, Tokyo, Japan

**Keywords:** IFN-γ, γδ T cells, malaria, dendritic cells, αβ T cells, memory cells, hematopoiesis

## Abstract

Malaria is caused by infection with *Plasmodium* parasites. Various studies with knockout mice have indicated that IFN-γ plays essential roles in protective immunity against blood-stage *Plasmodium* infection. However, after *Plasmodium* infection, increased IFN-γ production by various types of cells is involved not only in protective immunity, but also in immunopathology. Recent reports have shown that IFN-γ acts as a pro-inflammatory cytokine to induce not only the activation of macrophages, but also the generation of uncommon myelolymphoid progenitor cells after *Plasmodium* infection. However, the effects of IFN-γ on hematopoietic stem cells and progenitor cells are unclear. Therefore, the regulation of hematopoiesis by IFN-γ during *Plasmodium* infection remains to be clarified. Although there are conflicting reports concerning the significance of γδ T cells in protective immunity against *Plasmodium* infection, γδ T cells may respond to infection and produce IFN-γ as innate immune cells in the early phase of blood-stage malaria. Our recent studies have shown that γδ T cells express CD40 ligand and produce IFN-γ after *Plasmodium* infection, resulting in the enhancement of dendritic cell activation as part of the immune response to eliminate *Plasmodium* parasites. These data suggest that the function of γδ T cells is similar to that of NK cells. Although several reports suggest that γδ T cells have the potential to act as memory cells for various infections, it remains to be determined whether memory γδ T cells are generated by *Plasmodium* infection and whether memory γδ T cells can contribute to the host defense against re-infection with *Plasmodium*. Here, we summarize and discuss the effects of IFN-γ and the various functions of γδ T cells in blood-stage *Plasmodium* infection.

Malaria is one of the most serious public health problems worldwide, and the malaria-endemic areas include both tropical and subtropical regions. *Plasmodium*, the malaria-causing protozoan parasite, has a complex life cycle. Mammalian hosts, such as humans, monkeys, and mice, are inoculated with sporozoites into the host dermis of skin via a bite by a *Plasmodium*-infected *Anopheles* mosquito (1). Sporozoites then enter blood stream for invasion into hepatocytes and develop into merozoites with replication (liver-stage). Merozoites parasitize red blood cells (RBCs), replicate, and then rupture the RBCs, allowing for new infection of additional normal RBCs (blood-stage). In contrast to asymptomatic liver-stage malaria, blood-stage malaria is the symptomatic phase. Thus, protective immunity against blood-stage malaria is important for reducing the severity of disease (Figure 1).

**Figure 1:**
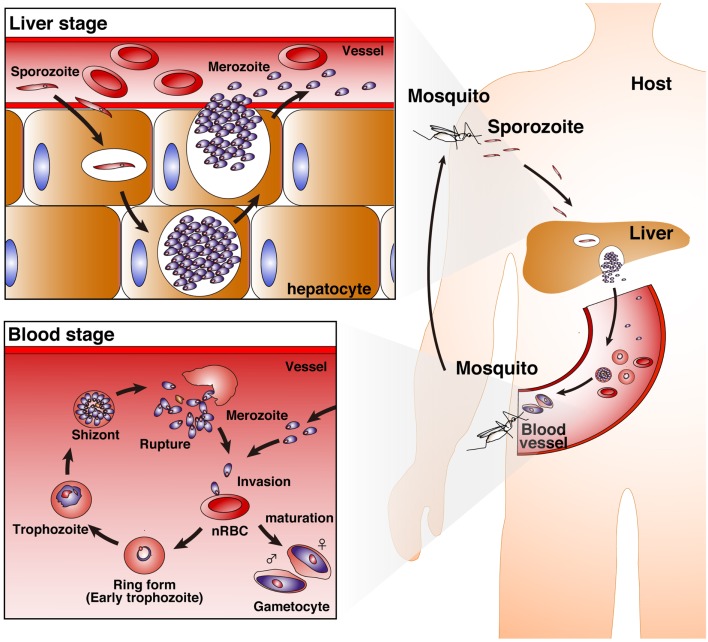
**Life cycle of *Plasmodium* parasites**. A bite from a *Plasmodium*-infected *Anopheles* mosquito inoculates human hosts with sporozoites in the dermis of skin. The sporozoites move into the bloodstream, and parasitize hepatocytes. Invaded sporozoites develop into merozoites with replication (liver-stage). Then, merozoites are released from hepatocytes. Merozoites parasitize red blood cells (RBCs), replicate, and then rupture the RBCs, leading to new infection of normal RBCs (nRBCs; blood-stage). Merozoites have several developmental phases: the ring form (early trophozoite), trophozoite, and schizont. Furthermore, some merozoites mature into female and male gametocytes.

Infection with *Plasmodium falciparum* causes the most severe malaria in humans. The predominant symptoms are anemia, splenomegaly, and fever. Cerebral malaria, liver dysfunction, acute renal failure, acidosis, hypoglycemia, respiratory distress, and edema are also observed as complications in malaria patients, although these symptoms do not always appear. Certain *Plasmodium* species can specifically infect rodents and cause malaria. A rodent malaria model is very useful not only for the development of anti-malarial drugs and vaccines, but also in research studies into the protective and pathologic immune responses during malaria. The lethality of the *Plasmodium* infection is dependent upon combinations of *Plasmodium* species and inbred mouse strains. Here, we review experimental studies on *Plasmodium* infections in the mouse and human studies with *P*. *falciparum*, focusing on protective immunity against *Plasmodium* infections.

Interferon-γ (IFN-γ) is produced mainly by lymphocytes, such as αβ T cells, natural killer (NK) cells, NKT cells, and γδ T cells (2–5). Some myeloid cells have also been reported to have the potential to produce IFN-γ (6–10). IFN-γ is an important pro-inflammatory cytokine and a mediator of immune responses against intracellular bacteria and viruses (11–14). Furthermore, it plays a protective role against infection by protozoan parasites (15–18). It enhances phagocyte activity, resulting in the elimination of extracellular bacteria and protozoan parasites, and its production by CD4^+^ helper T cells, CD8^+^ killer T cells, and NK cells is greatly induced by IL-12 and IL-18 from antigen-presenting cells (APCs), such as dendritic cells (DCs) (19–22). Moreover, some reports have shown that IFN-γ production from APCs is regulated by IL-12, IL-15, and IL-18 (23–25).

γδ T cells work as innate lymphocytes, the first line of defense against infectious pathogens. On the other hand, αβ T cells, such as CD4^+^ helper T cells and CD8^+^ killer T cells, are typically related to adaptive immunity. γδ T cells play critical roles in protective immune responses against protozoan parasites, bacteria, and viruses that are associated with various infectious diseases (26–32). This review focuses on the protective abilities of γδ T cells and IFN-γ in the response against malaria infection.

## IFN-γ Mediates Protective Immunity Against Blood-Stage *Plasmodium* Parasites

**Figure 2:**
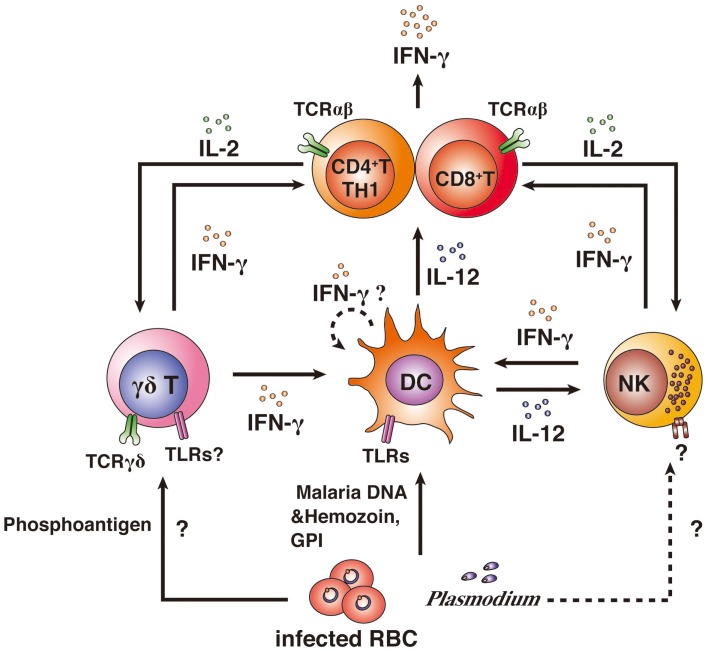
**IFN-γ producers and their activation**. CD4^+^ and CD8^+^ αβ T cells, NK cells, and γδ T cells have been shown to produce IFN-γ after infection with *Plasmodium* parasites. Innate immune cells can directly recognize *Plasmodium* antigens. γδ T cells can recognize phosphoantigens by MHC II-independent pathways *via* TCRγδ, resulting in the activation of DCs and IFN-γ production. Whether γδ T cells can recognize malarial antigens *via* TLR is unknown. DCs phagocytose iRBCs and recognize malarial DNA and hemozoin or glycosylphosphatidylinositol (GPI) anchors *via* TLRs, resulting in the activation of DCs. DCs are thought to have the potential to produce IFN-γ. However, it remains unclear as to whether IFN-γ production from DCs is induced by malarial antigens. Activated DCs produce IL-12 and present antigens to αβ T cells. αβ T cells are strongly activated by IL-12 and antigen presentation. IL-12 also activates NK cells. The malarial antigen-recognition receptor on NK cells remains to be determined. Activation of αβ T cells leads to high IFN-γ production. Furthermore, activated αβ T cells produce IL-2, resulting in the proliferation of γδ T cells and NK cells and the αβ T cells themselves.

Mice (on a C57BL/6 or CBA background) that are genetically IFN-γ-deficient or IFN-γ receptor (IFN-γR)-deficient or that are treated with anti-IFN-γ antibody and infected with blood-stage *P*. *berghei* are unable to control the infecting parasite (33–35). In the cases of infection with blood-stage *P*. *chabaudi* and *P*. *yoelii* parasites, genetically IFN-γ-deficient or IFN-γ receptor (IFN-γR)-deficient mice or anti-IFN-γ antibody-treated mice on a C57BL/6 or CBA background show delayed elimination of the parasites (36–39). These experimental malaria models demonstrate that IFN-γ is a key pro-inflammatory cytokine for controlling blood-stage *Plasmodium* parasites (Table 1). IFN-γ is produced by many cell types and involved in many steps of immune responses. αβ T cells, NK cells, NKT cells, and γδ T cells have been shown to produce IFN-γ after infection with *Plasmodium* parasites. The contributions of producers of IFN-γ to protective immunity against *Plasmodium* parasites are complicated (Figure 2). Thus, more detailed experiments using IFN-γ signaling-deficient models should be performed to determine the mechanism(s) underlying the involvement of IFN-γ in immune protection against *Plasmodium* parasites.

**Table 1:** **Influence of IFN-***γ***-signal deficiency on control of *Plasmodium* parasites in mice**.

*Plasmodium* strain	Host mouse genotype (mAb administration)	Mouse background	Features of host mice after the *Plasmodium* infection	Reference
*P. yoelii* 17XNL	IFN-γ KO	B6/129	Delayed elimination of parasites and had higher parasitemia	van der Heyde et al. (37)
*P. chabaudi adami* 556KA	IFN-γ KO	B6/129	Delayed elimination of parasites and had higher parasitemia	van der Heyde et al. (37)
*P. chabaudi chabaudi* AS	IFN-γR KO IFN-γ KO	B6/129 B6	Higher parasitemia during second peak. 77% of the infected mice died Male mice developed higher parasitemia. Female mice delayed elimination of parasites and had higher parasitemia. 100% Male and 40% female mice died	Favre et al. (36), Su and Stevenson (39)
*P. berghei* XAT	WT (anti-IFN-γ mAb) IFN-γ KO	CBA B6	Could not eliminate parasites and died	Waki et al. (33), Yoneto et al. (35)
*P. berghei* NK65	WT (anti-IFN-γ mAb) WT (anti-IFN-γ mAb)	CBA B6	*P. berghei* NK65 is a high virulent strain and induces liver injury in CBA and B6 mice. The mAb-treated mice prolonged survival	Waki et al. (33), Yoshimoto et al. (34)
*P. berghei* ANKA	IFN-γR KO IFN-γ KO IFN-γR KO	CBAB6B6/129	*P. berghei* ANKA is a high virulent strain and induces neurological symptoms in 129 and B6 mice. The KO mice prolonged survival and did not developed neurological symptoms	Amani et al. (38), Villegas-Mendez et al. (61), Rudin et al. (94)

IFN, interferon; IFN-γR, IFNγ receptor; mAb, monoclonal antibody.

## IFN-γ Producers and Their Activation in *Plasmodium* Infection

### CD4^+^ αβ T cells and CD8^+^ αβ T cells

Given that MHC class II-deficient mice on a C57BL/6 background are unable to control *P*. *yoelii-* and *P*. *chabaudi*-infected RBCs (iRBCs), CD4^+^ αβ T cells respond to *Plasmodium*-iRBCs after priming with malarial antigens in an MHC class II context on DCs (40). CD4^+^ αβ T cells strongly increase their ability to produce IFN-γ after infection with *P*. *berghei* (32). High proportions of CD4^+^ αβ T cells from *P*. *falciparum*-infected human subjects respond to iRBCs and produce IFN-γ, compared to CD4^+^ αβ T cells from naïve human subjects (4). Moreover, some studies have shown that CD4^+^ αβ T cell-depleted C57BL/6 or CBA mice treated with anti-CD4 antibody are unable to control blood-stage *P*. *chabaudi* or *P*. *berghei* (32, 33, 41, 42). These lines of evidence suggest that CD4^+^ αβ T cells are major sources of IFN-γ and are required for protective immunity against *Plasmodium* infection, resulting in complete clearance of the parasites. In contrast, the results for *Plasmodium*-infected mice with CD8^+^ αβ T-cell depletion are comparable to those for control mice (33, 41, 42). Moreover, CD8^+^ αβ T cells from highly immunized mice may play critical roles in protective immunity against infection with a lethal *P*. *yoelii* strain in C57BL/6 mice (41–43), although CD8^+^ T cells are not required for the resolution of primary infection with *P*. *chabaudi* or *P*. *yoelii* parasites in C57BL/6 mice. Interestingly, involvement of perforin molecules is only partial in protective immunity involving CD8^+^ αβ T cells. IFN-γ and macrophages are key molecular and cellular factors in protective immunity involving CD8^+^ αβ T cells (43). These data suggest that the role of the CD8^+^ αβ T cells is redundant in relation to the role of CD4^+^ αβ T cells in protective immunity in repeatedly immunized mice. A repeat infection with *Plasmodium* parasites may induce CD4^+^ αβ T-cell exhaustion (44). Cytokine production by T cells is inhibited by this exhaustion *via* the signaling of inhibitory receptors, such as PD-1 and LAG3. Since CD4^+^ αβ T cells are still needed to exert protective immunity against re-infection with the *Plasmodium* parasites, only partial exhaustion of CD4^+^ αβ T cells may occur. Therefore, both CD4^+^ αβ T cells and CD8^+^ αβ T cells may need to produce adequate quantities of IFN-γ to ensure clearance of the *Plasmodium* parasites.

### Dendritic cells

Several reports have shown that IFN-γ is also produced by DCs (8–10). The IFN-γ-producing DCs are important for priming lymphocytes (10), although it remains to be determined whether IFN-γ production by DCs enhances protective immunity against *Plasmodium* parasites, and whether stimulation of Toll-like receptors (TLRs) by parasite components induces IFN-γ production by DCs (45–48).

### γδ T cells and NK cells

γδ T cells and NK cells are considered to be important IFN-γ producers in blood-stage malaria infections and to be associated with the control of malarial parasites (2, 3, 5). In the early stages of malaria infection, γδ T cells directly recognize the pathogen through MHC-independent mechanisms that involve the γδTCR, and high levels of IFN-γ production and proliferation are induced (49–51). Several reports have suggested that proliferation of γδ T cells depends on IL-2 (52, 53). Human and murine γδ T cells usually express TLRs, and the expression levels of TLRs are enhanced by γδTCR stimulation (54–56). Therefore, TLR is another candidate receptor for γδ T-cell responses to malaria antigens (46, 57). Although studies have suggested that γδ T cells have the potential to react to malarial antigens via TLRs, TLR signaling in myeloid cells may be more important for the induction of protective immunity against malaria (58).

A study of experimental *P. falciparum* infection showed that, in contrast to γδ T cells, NK cells were minor IFN-γ producers in response to iRBCs before and after *P. falciparum* infection (5). A longitudinal study of children in Papua New Guinea indicated that IFN-γ-producing responses of malaria antigen-specific γδ T cells, but not those of NK cells, were important for protective immunity against *P. falciparum* infection (59). In contrast, several *in vitro* culture studies have shown that NK cells rapidly induce IFN-γ production in response to *P. falciparum*-iRBCs *via* IL-12 and IL-18 signaling. These reports suggest that NK cells have the potential to produce IFN-γ in response to *P. falciparum*.

## IFN-γ Induces Pathologic Effects in Severe Malaria

Interferon-γ is not only a key factor in protection against *Plasmodium* infection, but also a pathogenicity factor in severe malaria symptoms (33, 38, 60, 61, 94). It is well-known that *P*. *berghei* ANKA infection leads to the development of experimental cerebral malaria (ECM), and *P. berghei* NK65 infection induces liver injury. Those *P*. *berghei*-infected mice are useful models for studies of severe malarial symptoms. IFN-γR-deficient mice on a C57BL/6 or 129 background do not develop ECM. Furthermore, *P. berghei* NK65-infected mice on a C57BL/6 or CBA background treated with anti-IFN-γ antibody live longer than those treated with control-IgG. These reports suggest that IFN-γ is important for the development of severe malarial symptoms (Table 1) (33, 34, 38, 94). ECM also does not develop in *P*. *berghei* ANKA-infected mice after depletion of CD4^+^ T cells or CD8^+^ T cells (62). Cytotoxic CD8^+^ αβ T cells, which express perforin or granzyme B, have important roles in the development of ECM (63, 64). Although both CD4^+^ αβ T and CD8^+^ αβ T cells have a similar potential to produce IFN-γ, CD8^+^ αβ T cells are not an important IFN-γ producer for the development of ECM (61). IFN-γ-producing CD4^+^ αβ T cells promote recruitment of cytotoxic CD8^+^ αβ T cells to the brain; in contrast, CD4^+^ αβ T cells dot not accumulate markedly in the brain (60). Because depletion of NK cells can prevent ECM after infection with *P*. *berghei* ANKA, NK cells are also associated with IFN-γ responses in the development of ECM. NK cells activate CD8α^+^ DCs that prime CD8^+^ αβ T cells after infection with *P*. *berghei* ANKA (65). Furthermore, depletion of γδ T cells can also prevent ECM after infection with *P*. *berghei* ANKA. Therefore γδ T cells would be also associated with IFN-γ responses in the development of ECM (66). IFN-γ has both protective and pathologic effects on the immune response to *Plasmodium* infections. Therefore, simply inducing a reduction in blood IFN-γ levels by treatment with an antibody or drug may not be an effective way to treat cerebral malaria. Further studies focused on the regulation of CD8^+^ αβ T-cell activation by DCs are needed to develop a preventative therapy for cerebral malaria.

## Does IFN-γ Induce Effective Hematopoiesis in the Host?

IFN-γ is an important mediator of hematopoietic stem cell and progenitor cell activation during bacterial infections (67–69). Furthermore, rodent *Plasmodium* infection induces the generation of uncommon myelolymphoid progenitor cells, which express IL7-R^+^ and c-Kit^hi^ *via* IFN-γ signaling. These myelolymphoid progenitor cells can differentiate into both myeloid cells and lymphoid cells (70). Host protection from malaria depends on hematopoietic differentiation (i.e., hematopoiesis) to supply many types of differentiated cells. First, differentiation of erythroid progenitor cells is necessary to avoid the development of severe anemia. Second, differentiation of myeloid progenitor cells is necessary to supply a high number of phagocytic cells for the clearance of *Plasmodium* parasites. Hematopoiesis is a fundamental process for “curing” malaria and other infections. Moreover, several studies have shown IFN-γ-induced inhibition of some steps of hematopoiesis (71–73). Considering important roles of IFN-γ in protective immunity against *Plasmodium* parasites, IFN-γ would be a key factor for regulation of effective hematopoiesis in malaria. There are many cell types of IFN-γ producers in *Plasmodium* infection as mentioned above. Therefore, some of the IFN-γ producers in *Plasmodium* infection may play crucial role for regulation of hematopoiesis by the effect of IFN-γ or a combination of IFN-γ and other factors, which are produced from the IFN-γ-producing cells.

## Host Immune Responses of γδ T Cells in Malaria

### Varieties of γδ T-cell function

γδ T cells play various roles, including in protective immunity against pathogens, in the curing of injured tissue, tumor surveillance, and as a bridge between innate and adaptive immunity. These multiple functions of γδ T cells are thought to be due to their abilities to produce various cytokines and chemokines. Such abilities are broadly restricted by the Vγ and Vδ repertoires of γδ T cells. The distribution of γδ T-cell subsets differs depending on their resident tissue (74). To examine the physiologic role(s) in the immune response against *Plasmodium* parasites, γδ T-cell subsets should be compared carefully between mice and humans based on their abilities, such as cytokine production or ligands for activation.

### γδ T-cell-related protective immunity against blood-stage *Plasmodium* infection

**Figure 3:**
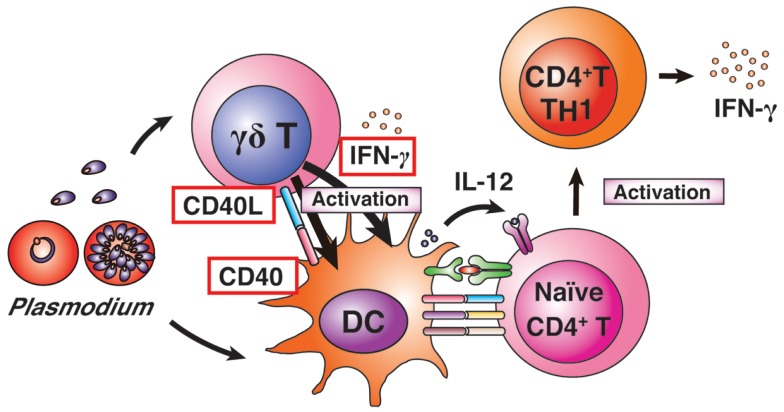
**γδ T-cell-related protective immunity**. γδ T cells and DCs are activated by *Plasmodium* infection. Activated γδ T cells begin to express CD40L and produce IFN-γ. The activated γδ T cells enhance MHC II and co-stimulatory factors on DCs, and induce high levels of IL-12 production from DCs. The CD40L-expressing γδ T cells enhance DC activation. Activated DCs induce differentiation of naïve CD4^+^ T cells into IFN-γ-producing T_H_1 cells.

Although previous reports of *in vitro* and *in vivo* experiments have suggested that γδ T cells are associated with protective immune responses during malaria infection, the functions of γδ T cells in the spleen remain largely unknown (75–79). We recently reported on the mechanism of γδ T-cell-related protective immunity against *Plasmodium* parasites using the rodent malaria parasite *P*. *berghei* XAT (32, 80) (Figure 3). *P*. *berghei* XAT is an attenuated strain derived from the lethal *P*. *berghei* NK65 strain (32, 81). *P*. *berghei* XAT was developed for investigations of vaccines and protective immune responses against *Plasmodium* parasites owing to its potent ability to induce immune memory, even against lethal *P. berghei* NK65 strains. Previous studies have reported the essential cytokines and immune cells required for the clearance of *P*. *berghei* XAT. Since γδ T-cell-deficient mice on a C57BL/6 background are unable to control *P*. *berghei* XAT infection, γδ T cells are essential for protective immunity against the parasites (32). Other studies using *P*. *chabaudi* parasites have also suggested that γδ T cells are related to, but not essential for, protective immunity against the parasites (76, 78). Thus, this *P*. *berghei* XAT strain is useful for investigating the mechanism(s) of γδ T-cell-related protective immunity against *Plasmodium* parasites. In general, the immunologic functions of γδ T cells are largely similar to those of NK cells. The difference in the need for γδ T cells between protective immunity against the two parasite strains may be influenced by the contribution of NK cells to protective immunity.

Although malaria infections in humans, monkeys, and mice lead to increased numbers of γδ T cells in the blood and spleen, the γδ T-cell population is still minor in relation to the total number of lymphocytes (82). It may be possible to explain why such a minor population of lymphocytes plays such key roles in protective immunity against malaria parasites. First, γδ T cells can directly recognize malarial parasites, making them major producers of IFN-γ in response to malaria antigens during the early phase of infection. In contrast, NK cells are reciprocally regulated by DCs. Thus, DC activation is needed first to activate NK cells in malaria infection (3, 83). NK cells are unexpectedly minor producers of IFN-γ in response to malarial antigens in human blood in experimental infections with *P*. *falciparum* parasites (59). Nevertheless, NK cells also become key players in protective immunity against malarial parasites, as has been shown in many studies (2, 3, 83), although NK cells are not required for the control of *P*. *berghei* XAT parasites (35). Second, γδ T cells have the ability to interact readily with other central immune players, such as DCs. Our recent report showed that about 30% of splenic γδ T cells were localized in the vicinity of DCs even under naïve conditions. A more than twofold increase in the percentage of splenic γδ T cells that adhered to DCs was observed in the early phase of infection (32). The molecular mechanism of this adhesive ability of γδ T cells to DCs remains to be determined. As γδ T cells can produce chemokines, some γδ T-cell-produced chemokines may attract DCs and also αβ T cells to help antigen presentation by DCs (84, 85). Furthermore, our study provided evidence that γδ T cells boost DC activation for protective immunity against *P*. *berghei* XAT parasites *via* CD40 ligand expression on γδ T cells. CD40L-CD40 signaling induces the expression of MHC II and co-stimulatory factors, such as CD40, CD80, and CD86 (32). Such signaling to DCs would be synergistically activated by the uptake of *Plasmodium* antigens (86).

### Memory phenotype of γδ T cells in malaria

**Figure 4:**
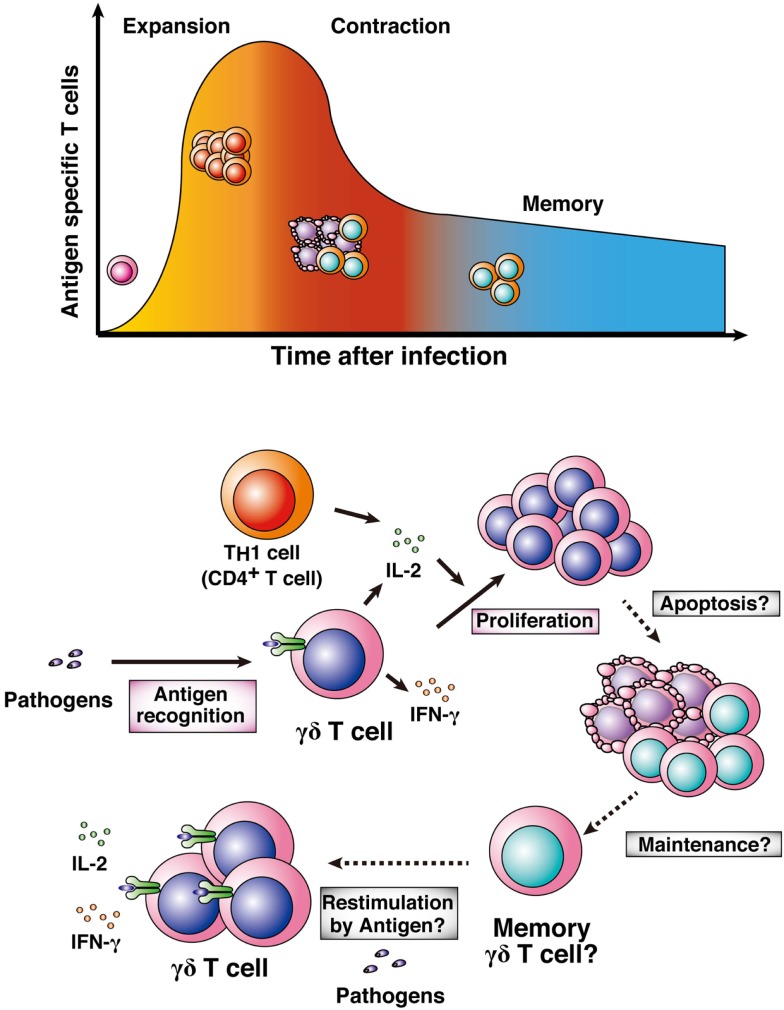
**Memory γδ T cells**. Naïve T cells are activated by pathologic infections and differentiate into effector or killer T cells. Activated T cells clonally expand in response to particular antigens (expansion phase). After pathogen clearance, high proportions of the expanded antigen-specific T cells undergo apoptosis (contraction phase). Some population of the surviving antigen-specific T cells is maintained as memory T cells (memory phase). γδ T cells also expand after infection with pathogens, including *Plasmodium* parasites. High-level proliferation of γδ T cells is involved in generating IL-2 from T_H_1 cells or the γδ T cells themselves. It remains to be determined whether antigen-specific γδ T cells shift into a contraction phase and then a memory phase.

For long-standing protective immunity against pathogens, memory lymphocytes maintain phenotypes to respond against pathogens and live for a long period after infections or vaccination with antigens (87, 88). In general, memory T cells are converted from naïve T cells through three phases (expansion, contraction, and memory phases; Figure 4). In the expansion phase, effector or killer T cells activate and proliferate after pathologic infection. Soon after pathogen clearance, the numbers of expanded antigen-specific T cells are reduced by induction of apoptosis in the contraction phase. Then, a fraction of the remaining antigen-specific T cells become memory T cells, resulting in long-lasting and rapid protective immunity against re-infection with pathogens (memory phase). Memory plasma cells and B cells maintain the potential to generate antigen-specific antibodies, and memory CD4^+^ T cells and CD8^+^ T cells maintain rapid helper and killer responses to antigens, resulting in rapid elimination of re-infecting pathogens. In malaria, those memory lymphocytes would also exist in human subjects who have been infected with *Plasmodium* parasites previously (4, 5). However, there is still controversy over the duration of memory lymphocyte maintenance (89, 90). Previous studies have shown that human γδ T cells generate rapid and amplified responses to a secondary challenge of bacteria and viruses, such as mycobacteria and cytomegalovirus (91, 92). These data suggest that human γδ T cells have the ability to develop memory cells for protection against re-infection. On the other hand, a recent report suggested that human γδ T cells develop effector memory cells after infection with *P*. *falciparum* parasites (5).

Some details about memory γδ T cells in malaria remain to be uncovered (Figure 4). The first point is the importance of memory γδ T cells in malaria. Although the numbers of malaria antigen-specific γδ T cells certainly increase in the year after primary infection, whether the memory γδ T cells are important for effective protection against re-infection with *Plasmodium* parasites remains to be determined. The second point is the developmental process of memory γδ T cells. γδ T cells function not only in adaptive immunity but also in innate immunity. Thus, the process for the development of memory γδ T cells may differ from that of general memory T cells in malaria or in other infections. The third point is the localization of memory γδ T cells. A recent report showed that memory T cells in lymph nodes and peripheral tissues translocate to bone marrow for the long term (93). It would be difficult to examine whether this hypothesis applies to memory γδ T cells, and other memory lymphocytes, in human subjects. Thus, rodent malaria models are needed for examining the location of memory γδ T cells.

## Concluding Remarks

Many studies have demonstrated that IFN-γ is responsible for protective immunity against *Plasmodium* parasites. The critical cellular source(s) of IFN-γ in malaria patients remains to be determined. γδ T cells are not only a major candidate as the key IFN-γ producer, but are also critical modulators of protective immunity against *Plasmodium* parasites. We should continue to investigate the roles of γδ T cells in host defenses against *Plasmodium* parasites. Thus, it is important to investigate whether the abilities of γδ T cells to produce IFN-γ and mediate immune responses against malarial antigens persist as memory cells. Mechanisms of immune responses in lymphoid tissues, especially in the spleen, to blood-stage malaria are gradually being uncovered by studies using rodent malaria models. To investigate further the relationship between IFN-γ and γδ T cells in immune responses against malaria, we should make more effort to study the issues using not only rodent malaria models, but also humanized mice that can be infected with *P*. *falciparum* parasites. To make this a reality, we first need to refine the humanized mice. Based on studies of γδ T-cell-related protective immunity, we may be able to develop novel strategies for a malaria vaccine.

## Conflict of Interest Statement

The authors declare that the research was conducted in the absence of any commercial or financial relationships that could be construed as a potential conflict of interest.
